# In vitro oxygen imaging of acellular and cell-loaded beta cell replacement devices

**DOI:** 10.1038/s41598-023-42099-w

**Published:** 2023-09-20

**Authors:** Mrignayani Kotecha, Longhai Wang, Safa Hameed, Navin Viswakarma, Minglin Ma, Cherie Stabler, Corinne A. Hoesli, Boris Epel

**Affiliations:** 1 Oxygen Measurement Core, O2M Technologies, LLC, Chicago, IL 60612 USA; 2 Department of Biological and Environmental Engineering, Cornell University, NY, 14853 USA; 3 Department of Biomedical Engineering, University of Florida, Gainesville, FL 32611 USA; 4 Department of Chemical Engineering, McGill University, Montreal, QC H3C 0C5 Canada; 5 Department of Radiation and Cellular Oncology, The University of Chicago, Chicago, IL 60637 USA

**Keywords:** Implants, Magnetic resonance imaging

## Abstract

Type 1 diabetes (T1D) is an autoimmune disease that leads to the loss of insulin-producing beta cells. Bioartificial pancreas (BAP) or beta cell replacement strategies have shown promise in curing T1D and providing long-term insulin independence. Hypoxia (low oxygen concentration) that may occur in the BAP devices due to cell oxygen consumption at the early stages after implantation damages the cells, in addition to imposing limitations to device dimensions when translating promising results from rodents to humans. Finding ways to provide cells with sufficient oxygenation remains the major challenge in realizing BAP devices’ full potential. Therefore, in vitro oxygen imaging assessment of BAP devices is crucial for predicting the devices’ in vivo efficiency. Electron paramagnetic resonance oxygen imaging (EPROI, also known as electron MRI or eMRI) is a unique imaging technique that delivers absolute partial pressure of oxygen (pO_2_) maps and has been used for cancer hypoxia research for decades. However, its applicability for assessing BAP devices has not been explored. EPROI utilizes low magnetic fields in the mT range, static gradients, and the linear relationship between the spin–lattice relaxation rate (R_1_) of oxygen-sensitive spin probes such as trityl OX071 and pO_2_ to generate oxygen maps in tissues. With the support of the Juvenile Diabetes Research Foundation (JDRF), an academic-industry partnership consortium, the “Oxygen Measurement Core” was established at O2M to perform oxygen imaging assessment of BAP devices originated from core members’ laboratories. This article aims to establish the protocols and demonstrate a few examples of in vitro oxygen imaging of BAP devices using EPROI. All pO_2_ measurements were performed using a recently introduced 720 MHz/25 mT preclinical oxygen imager instrument, JIVA-25™. We began by performing pO_2_ calibration of the biomaterials used in BAPs at 25 mT magnetic field since no such data exist. We compared the EPROI pO_2_ measurement with a single-point probe for a few selected materials. We also performed trityl OX071 toxicity studies with fibroblasts, as well as insulin-producing cells (beta TC6, MIN6, and human islet cells). Finally, we performed proof-of-concept in vitro pO_2_ imaging of five BAP devices that varied in size, shape, and biomaterials. We demonstrated that EPROI is compatible with commonly used biomaterials and that trityl OX071 is nontoxic to cells. A comparison of the EPROI with a fluorescent-based point oxygen probe in selected biomaterials showed higher accuracy of EPROI. The imaging of typically heterogenous BAP devices demonstrated the utility of obtaining oxygen maps over single-point measurements. In summary, we present EPROI as a quality control tool for developing efficient cell transplantation devices and artificial tissue grafts. Although the focus of this work is encapsulation systems for diabetes, the techniques developed in this project are easily transferable to other biomaterials, tissue grafts, and cell therapy devices used in the field of tissue engineering and regenerative medicine (TERM). In summary, EPROI is a unique noninvasive tool to experimentally study oxygen distribution in cell transplantation devices and artificial tissues, which can revolutionize the treatment of degenerative diseases like T1D.

## Introduction

Type-1 Diabetes (T1D) is an autoimmune disease that affects about ~ 1.5 million people in the USA and about 20 million worldwide, representing 5–10% of diagnosed cases of diabetes^1,2^. Islet transplantation can reverse hypoglycemia in most recipients for at least 1 year, demonstrating that cellular therapies can be curative if chronic immunosuppression can be circumvented and if adequate beta cell sources can be identified^2–5^. With progress towards robust beta cell production from pluripotent stem cells, avoiding graft rejection through genetic engineering^6–12^ or immunoisolating devices^13,14^ may become the bottleneck in diabetes cellular therapy. In both cases, encapsulation devices will likely be required to allow graft localization, retrieval, and hence safety.

A major requirement in this quest is to address the oxygen needs of glucose-sensitive insulin-producing islet cells. Islets are highly metabolically active and require adequate oxygenation for normal function. A higher oxygen consumption rate has been linked to better islet performance in BAP devices^3,15–19^. Unfortunately, in the absence of sufficient oxygen delivery, high consumption may lead to hypoxia, and many islets are lost during the isolation, encapsulation, and processing before the transplantation. Even more, islets are lost during the early engraftment period leading to the eventual failure of the device. Many approaches have been utilized to address the oxygen needs of islets^3,13,15,19–28^. These are: (a) maximizing the surface-to-volume ratio of devices for higher access to oxygen and nutrients, for example, using hollow fiber technology, flat sheet technology, microbeads encapsulation, etc.^3,29–31^, (b) oxygen generation in situ, typically using metal peroxides, for example, inverse breathing encapsulation device (iBED) that uses metal peroxide such as lithium peroxide (Li_2_O_2_) or OxySite that uses calcium peroxide (CaO_2_)^22,25,32–34^, and (c) stimulating oxygen delivery by promoting vascularization, developing modular assembly, using 3D printing technology to create a vascular structure^35–38^.

Regardless of the method of addressing the oxygen needs of islets, all devices will benefit from three-dimensional oxygen mapping because of oxygen distribution heterogeneity and the three-dimensional nature of these devices. With the support of the Juvenile Diabetes Research Foundation (JDRF), we established an “Oxygen Measurement Core” facility at O2M to perform oxygen measurements of BAP devices. This is an industry-academia partnership project. This article reports the first part of the project showcasing the capabilities of EPROI for providing in vitro pO_2_ imaging of BAP devices.

**Figure 1:**
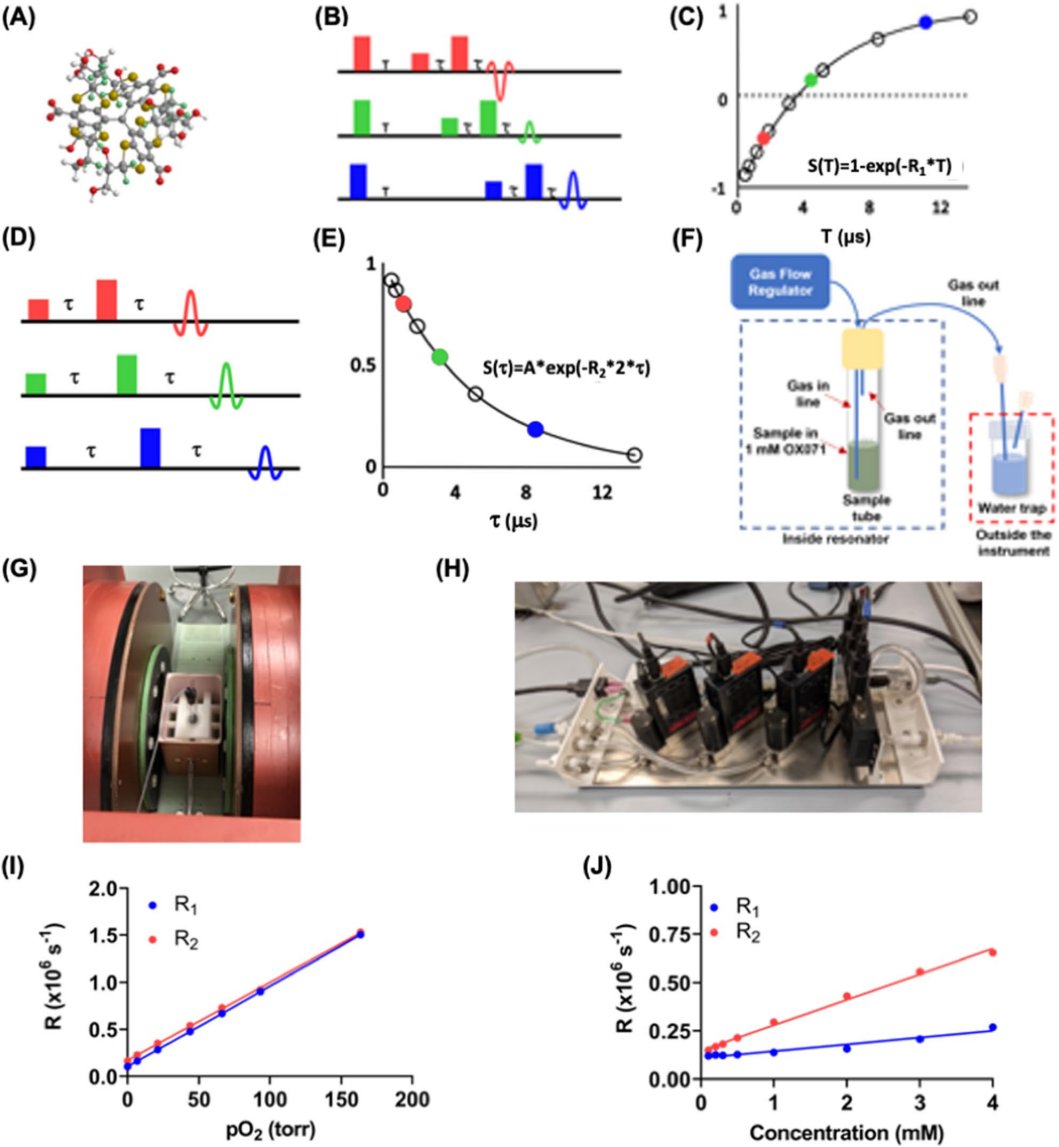
(**A**) The ball and stick model of Trityl OX071 chemical structure, (**B**) the inversion recovery electron spin echo (IRESE) pulse sequence used in this study to measure T_1_ (**C**) the monoexponential fit of the signal as a function of delay provides T_1_, (**D**) the electron spin echo (ESE) sequence used for T_2_ measurement, (**E**) a monoexponential fit for calculating T_2_, (**F**) the calibration setup used in this study, (**G**) an image showing a sample inside the 10 mm resonator used for calibration experiments, (**H**) the three channel mass flow controller for calibration set up, typically two channel air and N_2_ is sufficient for pO_2_ calibration, the third channel is used when a special gas combination is needed, f.e. 5% CO_2_ in case of iBED device measurements, (**I**) an example change in R_1_ (= 1/T_1_) and R_2_ (= 1/T_2_) as a function of pO_2_, the sample is PBS at 37 ºC, (**J**) the change in R_1_ and R_2_ as a function of trityl concentration at 0% oxygen, the sample is PBS at 37 ºC. Note that R_2_ had higher concentration dependence compared to R_1_, and therefore, R_1_ is used for pO_2_ measurements.

Electron paramagnetic resonance oxygen imaging (EPROI) is an established technique to obtain partial pressure of oxygen (pO_2_) maps in vitro and in vivo^39–41^. EPROI detects unpaired electron spins subjected to a static magnetic field, typically in mT range, by manipulating them using radio-frequency (200 MHz–1.1 GHz) electromagnetic radiation. Similar to magnetic resonance imaging (MRI), EPROI uses magnetic field gradients to generate images^42^. EPROI utilizes the linear relationship between pO_2_ and spin–lattice relaxation rate (R_1_) or spin–spin relaxation rate (R_2_) of oxygen-sensitive probes, such as trityl OX063 and its deuterated analog, OX071 (Fig. 1A)^43–45^. OX071 has a twice narrower linewidth that leads to an about twice higher signal-to-noise (SNR) ratio than OX063 for EPROI and is, therefore, a preferred spin probe for EPROI applications^46^. OX071 is highly soluble in water and can be added at the desired concentration to the sample for in vitro measurements^34,38^. Both R_1_ and R_2_ are linearly proportional to pO_2_. However, it has been shown using a 9 mT/250 MHz instrument that R_1_ is nearly independent of trityl concentration, which improves the accuracy of oxygen measurements^40^. The inversion recovery electron spin echo (IRESE) pulse sequence (Fig. 1B) that provides R_1_ via a single exponential fit (Fig. 1C) combined with the filtered back projection (FBP) reconstruction method is shown to deliver pO_2_ maps with the best accuracy and precision^47^. Over the past decades, EPROI has advanced at a rapid pace, from a continuous wave to pulse mode acquisition and improvement in imaging speed from 45 seconds to 1 min^33,40,41,47,48^. To date, the major application focus of EPROI has been in the preclinical (mostly mice) cancer hypoxia imaging^49–55^. Recently, many projects in the field of tissue engineering and regenerative medicine have been initiated by our group^33,38,56–59^.

In the current work, we extended the application of EPROI for the in vitro assessment of encapsulation devices developed for diabetes cellular therapy or bioartificial pancreas (BAP) devices. Our goal was to establish the framework and protocols for oxygen imaging of devices and perform the proof-of-concept pO_2_ imaging of five encapsulation devices originating from core members’ labs. These devices varied in size, shape, and biomaterial profile, and we established the compatibility of EPROI with these devices and developed the hardware, accessories, and protocols. This is the first such study to the best of our knowledge.

We have recently introduced a 25 mT preclinical pulse EPROI instrument, JIVA-25™, that operates at 720 MHz resonance frequency^60^. All pO_2_ imaging data were acquired using JIVA-25™. Since the 25 mT magnetic field has not been commonly used for EPROI, we started with the calibration of pO_2_ by performing R_1_ and R_2_ measurements of trityl OX071 for biomaterials used in the project. We also compared the EPROI accuracy to a commercially available point oxygen sensor OxyLite Pro in a few selected materials. Trityl OX063/OX071 have been reported to have very low in vivo toxicity (LD_50_ = 8 mmol/kg) and have been utilized for cancer hypoxia research since the early 2000s^39,41,55,61–64^. However, no data are available for its toxicity for cells that are commonly used in the T1D field. Therefore, we performed trityl OX071 toxicity studies using fibroblasts (as baseline model), as well as insulin-producing cells including beta-TC6, MIN6, and primary human islets. Finally, we performed oxygen imaging measurements of five acellular or cell-loaded BAP devices. These representative devices show a range of strategies commonly used in the field, and we demonstrate the utility of imaging these devices as compared to the single-point measurements commonly used in the field. Below, we present the results to make a case for using EPROI as a quality control tool in beta cell replacement therapies and other applications in the field of tissue engineering regenerative medicine.

## Materials and methods

### Trityl

Trityl OX071 (Tris(8-carboxyl-2,2,6,6-tetra(2-(1-hydroxy-2,2-*d*_2_-ethyl))benzo[1,2-*d*:4,5-*d′*]bis(1,3)dithiol-4-yl)methyl) in triacid powder form was purchased from N.N. Vorozthzov Novosibirsk Institute of Organic Chemistry of Siberian Branch of Russian Academy of Sciences (also available from O2M Technologies, LLC). The 72.2 mM trityl stock solution at neutral pH was prepared using the following method. The 1.0 g of trityl powder (MW: 1384.96) was mixed with 8.0 mL of deionized-double distilled water. The resulting suspension was titrated to pH 7.4 using 10 M, 1.0 M, and 0.1 M solutions of NaOH and HCl as needed, following which more deionized—double distilled water was added to a total volume of 10 mL. The neutral trityl stock solution was sterilized by filtering the liquid using a 0.22 μm syringe filter. The stock was stored at 4 °C. Before the experiments, the stock solution was diluted to the desired concentrations in media, buffer, or deionized water.

### Cells and cell culture supplies

Human fibroblasts (PCS-201-010), mouse beta-TC6 cells (Cat No: CRL11506) and Dulbecco's Modified Eagle's Medium (DMEM) (Cat No: 30-2002), Fibroblast Basal Medium (Cat No: PCS-201-030), Fibroblast Growth Kit-Serum-free (Cat No: PCS-201-040) were purchased from American Type Culture Collection (ATCC) (Manassas, Virginia). MIN6 cells were donated by Dr. Graeme Bell’s laboratory at the University of Chicago. DMEM with high glucose and pyruvate (Cat No: 11-995-065), 170 mM NaCl (BP310), 10 mM HEPES (S271) were purchased from FisherScientific. Fetal Bovine Serum (Cat No: 10082-147), Antibiotic–Antimycotic (100X) (Cat No: 15240062), Trypsin–EDTA (Cat No: 25200-056), PBS (pH 7.4: Cat No: 10010049) were purchased from ThermoFisher Scientific, Deionized-Double Distilled Water (Catalog # C993Y94) from Thomas Scientific, 0.22 μm syringe filter (SKU WHA9913-2502) from Millipore Sigma. ATPlite 1step Luminescence Assay System (Cat No: 6016736) was purchased from PerkinElmer (Downers Grove, Illinois).

### Biomaterials

Alginate powder (FMC Biopolymer) was a gift from Dr. Corinne Hoesli, McGill University, Canada, and Dr. Minglin Ma, Cornell University, New York. Chemicals were purchased from various companies: Glucono-δ-lactone (Cat No: G4750, Sigma-Aldrich), calcium carbonate (Avantor, 1301-01, fine grade particles), SeaPlaque agarose (Cat No: 50101, Lonza), agarose (Cat No: AG013, Sigma-Aldrich), gelatin (Cat No: G9391, Sigma-Aldrich). HEPES buffer saline (pH 7.4) was composed of 170 mM NaCl and 10 mM HEPES, sterilized by filtration (0.22 μm pore size).

### BAP devices

TheraCyte devices (20 μL) were purchased from TheraCyte, Inc., Laguna Hills, California. OxySite devices were prepared at O2M using the active and control disks (5 mm diameter × 1 mm thick PDMS disks with and without CaO_2_) provided by Dr. Cherie Stabler (University of Florida) according to the published protocol^25,32^. The iBED and SONIC devices were provided by Dr. Minglin Ma, Cornell University, New York^34,38^. Dr. Corinne Hoesli, McGill University, Canada, provided the single-channel macro-encapsulation device^65,66^.

### Trityl toxicity testing with cells

Cells (human fibroblasts, beta-TC6, MIN6, or Islets cells) were maintained in 100 mm culture dishes at 37 °C 5% CO_2_ in the incubator in DMEM supplemented with 10% FBS and 1% Antibiotic–Antimycotic. Cells were trypsinized with 0.05% trypsin–EDTA and were seeded to a 96-well plate in 100 µL cell culture medium. After overnight culture at 37 °C 5% CO_2_ incubator, cells were treated with trityl at various concentrations (0, 0.05, 0.1, 0.25, 0.5, 1.0, 4.0, 8.0 mM) in 100 µL cell culture media and incubated at 1-, 24-, and 48-h. Once incubation was over, spend media was aspirated out and cells were washed three times with cell culture media to remove trityl. Then 100 µL of cell culture medium was added to each well, followed by 100 µL substrate solution. Four replicates of each trityl concentration were prepared and used in the experiments. The toxicity was evaluated by measuring luminescence intensity using an ATPlite 1step Luminescence Assay kit that measures ATP, which is synthesized by viable cells only. Cells were incubated at room temperature for 15 min and luminescence was measured by Synergy LX Multi-Mode Reader (BioTek Instrument, Inc.).

### pO_2_ calibrations

Oxygen dependence of trityl relaxation in biomaterials was calibrated by equilibrating 1 mL of sample in a 10 mm test tube with a known oxygen concentration gas at atmospheric pressure. N_2_, CO_2_, air, and mixed-gas (5% CO_2_, 5% O_2_, 90% N_2_) cylinders were purchased from Medox, Inc. The desired O_2_ concentration was prepared using mass flow controllers MC-50SCCM-D/5M and MC-5SCCM-D/5M (Alicat Scientific). Dissolved gas equilibration for calibration was achieved by bubbling a gas mixture at a 6 sccm or higher flow rate. For low-viscosity liquid samples, a submerged silicone tube attached to the trocar of a 20 Ga IV catheter was used to bubble a sample. A vent tube containing another trocar of a 20 Ga IV catheter was used to keep the sample at atmospheric pressure. A third trocar of the same gauge was used to feed through a T-type thermocouple wire used as feedback to an air heating system, keeping the sample at the desired temperature (ranging from 24 to 37 °C). All trocars puncture through a rubber septum (10/30 joint), which provides the seal from an atmosphere. Hydrogel biomaterials require special treatment to achieve oxygen equilibration, as discussed in the following sections. Inversion recovery spectroscopy was performed as described in the “Oxygen imaging measurements” section. The resulting R_1_ values were correlated with the oxygen content of the delivered gas mixture, adjusted for local atmospheric pressure, temperature, and relative humidity.

OxyLite Pro was rented from Scintica Instrumentation, Inc and used according to the manufacturer’s instructions. OxyLite Pro is an optical fluorescence quenching device. The quenching rate is proportional to the pO_2_ in the vicinity of the dye. A custom septum was made for a 15 mm test tube using dental impression material in which a dedicated line was added for the OxyLite fiber optic probe.

### Gelatin

To prepare 1% gelatin by weight, 30 mg of dry gelatin was dissolved in 255 µL of deionized water in a 55 °C water bath. Once the gelatin was dissolved, an additional 2703.4 µL of deionized water was added to the tube, along with 41.6 µL of trityl stock solution (72.2 mM), resulting in a final volume of 3000 µL. The mixture was agitated until dissolved and immediately brought to the spectrometer for measurement, where it was kept at the desired temperature. At 37 °C, the gelatin mixture was allowed to equilibrate with gas mixtures. At room temperature, the gelatin is prone to foaming, an issue that can cause loss of sample over time. To collapse foam build-up, the sample was occasionally agitated.

### Agarose

A 2% agarose gel was prepared by mixing the low-melting-point agarose with PBS or media. The mixture was heated to 90 °C for 3 min and brought down to 40 °C before adding trityl. A 41.6 µL trityl stock solution (72.2 mM) was added to 2958.4 µL of gel to achieve the 1 mM trityl concentration for measurements. Agarose samples were gas equilibrated at approximately 40 °C and then allowed to gel before being measured with the desired gas circulating above the gelled sample at six sccm. A new agarose sample was made for each oxygen tension, as the previously gelled sample could neither be remelted in situ nor equilibrated once gelled.

### Alginate preparation

ALG1 (used for pO_2_ calibration) was prepared using 2.5% stock alginate mixed with 1.05% w/v CaSO_4_ suspension for a final concentration of 2% alginate. 100 mL of stock ALG1 was made by dissolving 2.5 g alginate powder into 90 mL of DMEM in a 500 mL medium bottle. The resulting mix was left stirring overnight at low speed or until the alginate was fully hydrated into the culture medium. Subsequently, 10 mL sterile-filtered fetal bovine serum was added to attain a 10% FBS concentration. Two mL gel sample was prepared in a 15 mm tube by combining 1.6 mL of 2.5% stock alginate with 0.4 mL CaSO_4_ suspension and 27.7 µL OX071 (for a final trityl concentration of 1 mM). The mixture was vigorously bubbled with N_2_ at 50 sccm before adding the calcium sulfate to achieve equilibration with N_2_ and overcome foaming issues. Calcium sulfate was added through a port made in the septum for an OxyLite Probe while N_2_ was circulated above the sample to prevent contamination with the atmosphere.

ALG2 (used in the macro-encapsulation device) was prepared using 2.5% alginate stock, as explained above but in HEPES buffered saline and without serum. The Ca^2+^ ions are supplied using calcium carbonate (CaCO_3_) suspension at a stock concentration of 0.5 M and released by lowering the pH using glucono-δ-lactone (GDL). The CaCO_3_ suspension was prepared by adding 1 g of CaCO_3_ powder to 20 mL HEPES buffered saline. Immediately before the preparation of any sample, the GDL solution was prepared by mixing 0.21396 g of GDL into 1 mL of HEPES buffered saline and vortexing the resulting mix until thoroughly dissolved. To maintain sterility, this preparation was sterile-filtered.

### OxySite with 2.5 million cells

Beta-TC6 cells in a 10 cm dish were trypsinized, counted to 2.5 million, and collected in a sterile 1.5 mL Eppendorf tube. 2 mL of 2% agarose gel (0.04 g in DMEM culture medium) was prepared in a glass tube by heating at 65 °C for 30 min. This was filtered using a 0.22 µm syringe filter. Alternatively, the glass tube containing 0.04 g agarose in 2 mL DMEM culture medium was heated to 90 °C for 5 min. Using a 1 mL pipet, 300 µL of agarose solution was aspired and mixed with the cell pellet. The mixture was quickly transferred to the mold, where the disk was fixed. The mold was placed in a fridge for 2 min for rapid gelation. The resulting construct was transferred to a cell culture tube with 2 mL DMEM containing 1 mM OX071.

### Inverse breathing encapsulation device (iBED) with 3.0 million fibroblasts cells encapsulated in 2% alginate gel

3.0 million fibroblasts were prepared and pelleted in a 15 mL sterile Falcon tube (1 million/mL) from a 100 mm culture dish. 2.4 mL 2.5% alginate solution (prepared in Fibroblast Culture Medium) was added to 20.7 µL stock trityl (OX071; final concentration: 0.5 mM). This was gently mixed with a 1 mL pipet to avoid the formation of bubbles. An iBED device was fixed at the bottom of the glass tube into cured dental impression material. The mixed alginate cell suspension with trityl was loaded into the glass tube using a pipette. Subsequently, 0.6 mL 1.05% (wt/vol) CaSO_4_ suspension was added (prepared in Fibroblast Culture Medium) into the alginate solution using a pipette and mixed immediately. 200 µL Fibroblast Culture Medium was added on top of the gelled sample to avoid drying during measurements. This sample was kept at 37 °C with an overhead flow of gas mixture (90% N_2_/5% CO_2_/5% O_2_) during pO_2_ measurement.

### TheraCyte (20 µL) with beta-TC6 cells, fibroblasts, or human islets

The cells from a 10 cm dish were trypsinized and counted for a harvest of 10 million or 5 million cells in a sterile 1.5 mL Eppendorf tube. Fibroblasts, 10 million, were treated with 70% alcohol for 30 min prior to use. The cells were loaded using a (70 µL) Hamilton syringe into a 20 µL TheraCyte device. The loading tube of the device was sealed with medical grade silicone adhesive (Factor II A100) using a 3 mL syringe with a 15 Ga luer stub adapter, and a piece of silicone tubing slipped over the loading port of the TheraCyte device. The port tube was then cut below the silicone tubing using a sterile scalpel. The device was then mounted using dental resin on the bottom of the 16 mm glass tube. 3 mL DMEM cell culture medium was added to the glass tube, followed by 41.6 µL of 72.2 mM OX071 stock solution (final trityl concentration is 1 mM). For the experiment with human islets, 10,000 IEQ islets were collected in a 1.5 mL tube (in 60 µL DMEM). The islet suspension was aspired in a 1.0 mL syringe without a needle and loaded through the port tube of a 20 µL TheraCyte device. Throughout the pO_2_ imaging experiment (24 h), an overhead flow of 95% air and 5% CO_2_ and 37 °C temperature was maintained.

### Perfused single-channel macro-encapsulation device (acellular or loaded with 24 million MIN6 cells)

To prepare 20 mL of alginate gel (ALG2) for the single-channel perfused macroencapsulation device, 16 mL of 2.5% stock alginate was mixed with 1.2 mL 0.5 M calcium carbonate suspension and 2 mL DMEM. To begin the gelation, 1 mL of freshly prepared GDL was added and stirred thoroughly with a spatula. The final concentrations for ALG2 were as follows: 2% alginate w/v, 30 mM CaCO_3_, and 60 mM GDL. Once GDL was added and the sample was mixed, complete gelation occurred within 30 min.

For preparing cell loaded perfused macro-encapsulation device, MIN6 cells cultured in 100 mm dishes were trypsinized, counted to 24 million, and collected in a sterile 1.5 mL Eppendorf tube as described previously^65,66^. To a sterile 50-mL beaker, 20 mL of 2.5% alginate stock solution, 346 µL 72.2 mM OX071 (1.0 mM final concentration), 2.0 mL DMEM, and 24 million MIN6 cells were added and mixed thoroughly using a sterile spatula. Next, 1.5 mL 0.5 M CaCO_3_ was added and mixed for one minute. 1.25 mL GDL (0.21396 g GDL in 1 mL HEPES buffered saline) was added and mixed for 1 min. The gel mixture was immediately loaded into a sterile macro-encapsulation device (which was prefixed in the resonator) using a 20-mL syringe and allowed to stand at room temperature for 30 min for gelation with a wire introduced into the device to form a channel. After gelation, the metal rod was removed to create a hollow channel and the device (containing 2% alginate gel with one mM trityl) was placed into the resonator and connected to a peristaltic pump (3 mL/min) and a sterile bottle containing DMEM with 10% FBS (60–80 mL) in a 37 °C incubator using silicone tubing (segments inside the resonator) or BPT tubing (segments outside the incubator) to form a circulation loop. The temperature was kept at 37 °C during the measurement.

### Oxygen imaging measurements

All pO_2_ maps were obtained using the JIVA-25™ (O2M Technologies, LLC) at the JDRF-supported “Oxygen Measurement Core” facility. Average pO_2_ measurements of the whole system (solution and device) were performed using inversion recovery with free induction decay detection spectroscopy sequence with the following parameters: pulse lengths 60 ns, 8 phase cycles, 80 logarithmically spaced delays from 350 ns to 40 μs, 55 µs repetition time. The curves were fitted using single exponential recovery to extract spin–lattice relaxation rates R_1_ (1/T_1_) values converted to pO_2_ (see: calibrations). The pO_2_ imaging was performed using IRESE sequence^67^ with the following parameters: pulse lengths 60 ns, 16 phase cycles, spin echo delay 500 ns, equal solid angle spaced 654 projections, 67 baselines, 1.5 G/cm gradient, eight logarithmically spaced time delays from 350 ns to 30 μs, 45 µs repetition time, overall, 10 min image duration. Images were reconstructed using filtered back-projection in isotropic 64 × 64 × 64 cube with 0.66 mm × 0.66 mm × 0.66 mm voxel size, 48 × 48 × 48 with 0.88 mm × 0.88 mm × 0.88 mm voxel size, or 32 × 32 × 32 cube with 1.33 mm × 1.33 mm × 1.33 mm voxel size.

## Results and discussion

The sections below describe the pO_2_ calibration procedure and provide data for the biomaterials used in this project at 25 mT using JIVA-25™ and a comparison of oxygen measurements with an OxyLite single-point probe for selected biomaterials. We also provide results of trityl toxicity studies. Finally, proof-of-concept in vitro pO_2_ imaging of five different BAP devices is demonstrated.

### Establishing oxygen imaging framework at 25 mT, pO_2_ calibration, and comparison with a single-point oxygen probe

EPROI experiments have been performed for cancer hypoxia studies since the early 2000s; however, no data or systematic study of EPROI in biomaterials exists. In addition, the previous EPROI experiments have been performed at 9 mT or 10 mT instruments. The relaxation rates R_1,_ R_2_ (= 1/T_1,_ 1/T_2_) of OX071 (Fig. 1A) are magnetic field and oxygen-dependent for constant salinity, temperature, and viscosity. Since a 25 mT instrument, along with many new biomaterials, was being used for the experiments in this study, we started with calibration experiments. As stated earlier, EPROI utilizes a linear relationship between the pO_2_ and the relaxation rates of trityl OX071 spins^40^. The T_1_ and T_2_ values at 0% oxygen are the maximum for a given sample, and it reduces as the oxygen increases. We performed R_1_ and R_2_ measurements of trityl OX071 at 37 °C. The R_1_ measurements were performed using the electron IRESE sequence (Fig. 1B), and the signal was fitted with a monoexponential recovery equation S(T) = 1 − exp (−R_1_*T) to obtain T_1_ (R_1_ = 1/T_1_) (Fig. 1C). R_2_ measurements were performed using an electron spin echo sequence as shown in Fig. 1D and fitting the signal with a monoexponential decay curve S(T) = A*exp (− R_2_*2*). to extract T_2_ (R_2_ = 1/T_2_) (Fig. 1E). Figure 1F shows the calibration setup that was used for the experiments. Briefly, trityl OX071 dissolved in the material of choice is loaded into a sample tube, and a gas mixture between 0 and 21% oxygen concentration is bubbled through the sample. Here, we used a 1 mL sample in a 10 mm sample tube (Fig. 1G) and performed all measurements in a 10 mm ID resonator. A setup with mass flow controllers (Fig. 1H) allows mixing air and N_2_ at a desired concentration to achieve the target O_2_ concentration for calibration purposes. The instrument heater control maintained the temperature at 37 ± 1 °C^68^.

Table 1A provides the T_1_ and T_2_ values for deionized water, PBS, DMEM media, DMEM media with cells, 1% gelatin, 2% agarose, and 1% alginate at 0% O_2_ and 37 °C.

**Table 1:** Calibration parameters for select biomaterials at 25 mT/720 MHz used in this project: (A) T_1_ and T_2_ at 37 °C and at 0% O_2_, (B) the intercept and the slope for a linear fit between R_1_ and pO_2_ (Fig. 1I).

A		
Biomaterial	T_1_ (μs)	T_2_ (μs)
PBS	7.75 ± 0.03	3.92 ± 0.01
DiH_2_O	8.79 ± 0.03	6.29 ± 0.01
DMEM media with 1 M cells	8.37 ± 0.02	4.05 ± 0.01
1% gelatin	7.23 ± 0.05	3.18 ± 0.01
2% agarose	7.98 ± 0.01	3.85 ± 0.01
1% alginate	8.18 ± 0.04	3.73 ± 0.03

B		
Biomaterial	Intercept (Ms^-1^)	Slope (torr/Ms^-1^)
PBS	0.1246	112.93
DiH_2_O	0.1138	108.42
DMEM media with 1 M cells	0.1195	114.05
1% gelatin	0.1191	114.13
2% agarose	0.1253	123.82
1% alginate	0.1223	119.58

The pO_2_ in a dissolved medium is calculated using Henry’s law. Henry’s law states that the concentration of dissolved gas is directly proportional to the partial gas pressure of that gas above the solution^69^:

1 $${p}_{a}={K}_{H}{C}_{W},$$

where ${p}_{a}$ is the partial pressure of the gas, ${C}_{W}$ is the concentration of gas in water, and ${K}_{H}$ is Henry’s law constant. K_H_ is a function of temperature.

The pO_2_ of the gas mixture that was supplied to the tube can be calculated as follows:

2 $${\text{Applied pO}}_{{2}} \, = \,\left( {{\text{atmospheric pressure}}{-}{\text{water vapor pressure}}} \right) \, *{\text{ oxygen concentration}}/{1}00.$$

Water vapor pressure was calculated assuming 50% of the humidity on the day of measurement.

3 $${\text{Calculated pO}}_{{2}} {\text{using EPR}}\, = \,\left( {{\text{R}}_{{1}} - {\text{intercept}}} \right) \, *{\text{slope}}$$

Figure 1I shows the change in R_1_ and R_2_ as a function of pO_2_ for 1 mM trityl in PBS at 37 °C as an example calibration curve. Both R_1_ and R_2_ are linearly proportional to pO_2,_ as expected. Figure 1J shows the change in R_1_ and R_2_ as a function of trityl concentration for PBS at 37 °C. It can be seen that R_2_ has a higher concentration dependence compared to R_1,_ in line with previously reported studies^40^. Therefore, R_1_ provides better oxygen accuracy and was used for all experiments in this study. R_1_ is proportional to the rate of collision between the unpaired electron spin of the trityl molecule and the O_2_ molecule. Therefore, it is a direct measure of the oxygen concentration in the sample. The pO_2_ values can be converted in mmol of O_2_ with 160 Torr (21% O_2_) = 0.0094 mol/L of O_2_. Note that OX071-EPROI provides pO_2_ measurement with high SNR and sensitivity in the range from 0 to 21% oxygen with high SNR and sensitivity under hypoxic conditions.

Table 1B provides the slope and intercept for R_1_ calibrations for deionized water, PBS, DMEM media, DMEM media with cells, 1% gelatin, 2% agarose, and 1% alginate at 37 °C and for 0%, 3%, 6%, 9%, 12%, and 21% O_2_ concentrations. All tested biomaterials show a linear correlation between pO_2_ and R_1_ values (Pearson coefficient very nearly equal to unity) with a linear dynamic range easily spanning full atmospheric oxygen tension (21% O_2_ or 160 torr) down to complete deoxygenation of samples.

Next, we compared EPROI with a commercially available fluorescent-based point sensor probe OxyLite Pro for a few selected materials. Table 2 shows the pO_2_ reading and errors from EPROI and OxyLite Pro compared to expected values at 37 °C for (A) deionized water, (B) 1% gelatin, and (C) 1 million MIN6 cells in DMEM medium for six oxygen conditions between 0 and 21%. As can be seen from Table 2, EPROI has higher accuracy of reporting pO_2_ from 0 to 21% O_2_ with increased accuracy at higher oxygen concentrations.

**Table 2:** Comparison of EPROI with a fluorescence-based single point probe for select biomaterials at 37 °C from 0 to 21% oxygen concentration.

A					
Supplied O_2_ (%)	Calculated theoretical pO_2_ (torr)	EPROI pO_2_ (torr)	Error with EPROI (torr)	OxyLite Pro pO_2_ (torr)	Error with OxyLite Pro (torr)
0	0.00	0.56	0.56	1.6	− 1.6
3	22.09	21.90	− 0.19	22	0.09
6	44.18	44	− 0.19	42.8	1.38
9	66.27	65.85	− 0.42	62.7	3.57
12	88.36	88.30	− 0.07	83	5.36
21	154.64	154.93	0.30	142.5	12.14

B					
Supplied O_2_ (%)	Calculated theoretical pO_2_ (torr)	EPROI pO_2_ (torr)	Error with EPROI (torr)	OxyLite Pro pO_2_ reading (torr)	Error with OxyLite Pro (torr)
0	0.00	2.21	2.21	1.7	− 1.7
3	22.59	22.74	0.15	22.1	0.49
6	45.17	44.41	− 0.76	42.6	2.57
9	67.76	66.01	− 1.75	63.5	4.26
12	90.34	88.49	− 1.85	83.7	6.64
21	158.10	160.11	2.01	144.7	13.40

C					
Supplied O_2_ (%)	Calculated theoretical pO_2_ (torr)	EPROI pO_2_ (torr)	Error with EPROI (torr)	OxyLite Pro pO_2_ reading (torr)	Error with OxyLite Pro (torr)
0	0.00	0.45	0.45	0.9	− 0.90
3	22.08	21.28	− 0.79	21.2	0.88
6	44.15	43.98	− 0.17	42.5	1.65
9	66.23	66.00	− 0.22	63.6	2.63
12	88.30	89.42	1.12	84	4.30
21	155.53	154.15	− 0.38	143.7	10.83

The desired concentration was achieved by bubbling the known mixture of air and N_2_ in the media (Fig. 1B) using a two-channel mass flow controller system, the second column represents the calculated pO_2_ using Eq. (2), the third and fifth columns represent the measured pO_2_ using EPROI and the fluorescence-based OxyLite Pro probe along with their errors from the expected values in the first column. The samples are: (A) de-ionized water, (B) 1% gelatin, (C) 1 million MIN6 cells in DMEM medium.

### Trityl OX071 is nontoxic to cells

**Figure 2:**
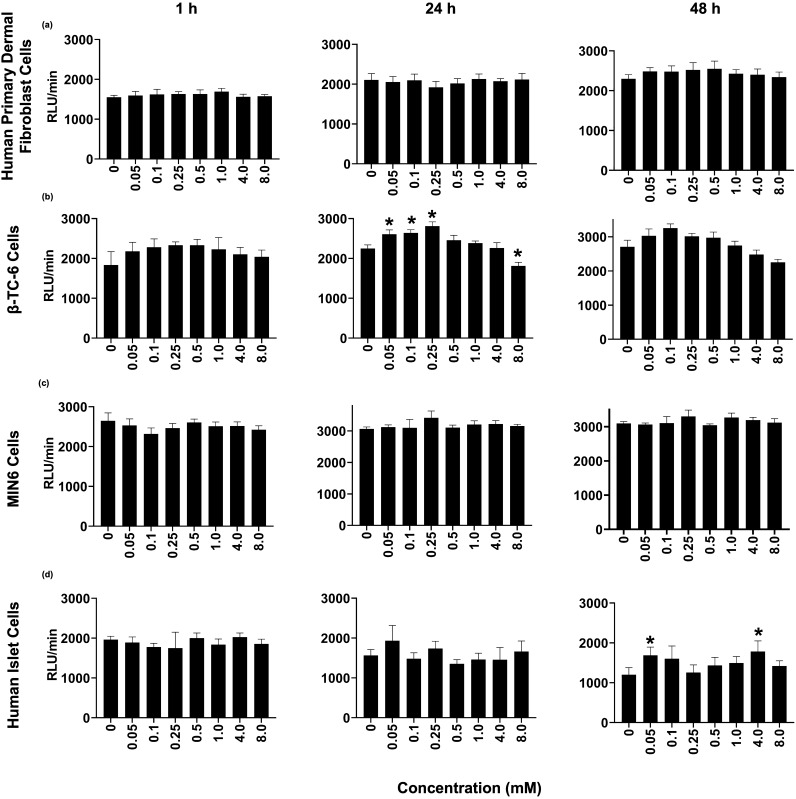
Luminescence intensity as a function of 8 different trityl concentrations (4 replicates  of each) at 1 h, 24 h, and 48 h of trityl treatment, (**a**) Fibroblasts at the density of 50,000/well, (**b**) Beta-TC6 Cells at the density of 80,000/well, (**c**) MIN6 cells at the density of 80,000/well, and (**d**) Human islets at the density 50 IEQ/well. Data are presented as mean ± SE. Statistical analysis was performed by student T-Test. *p < 0.05 vs. Control.

**Figure 3:**
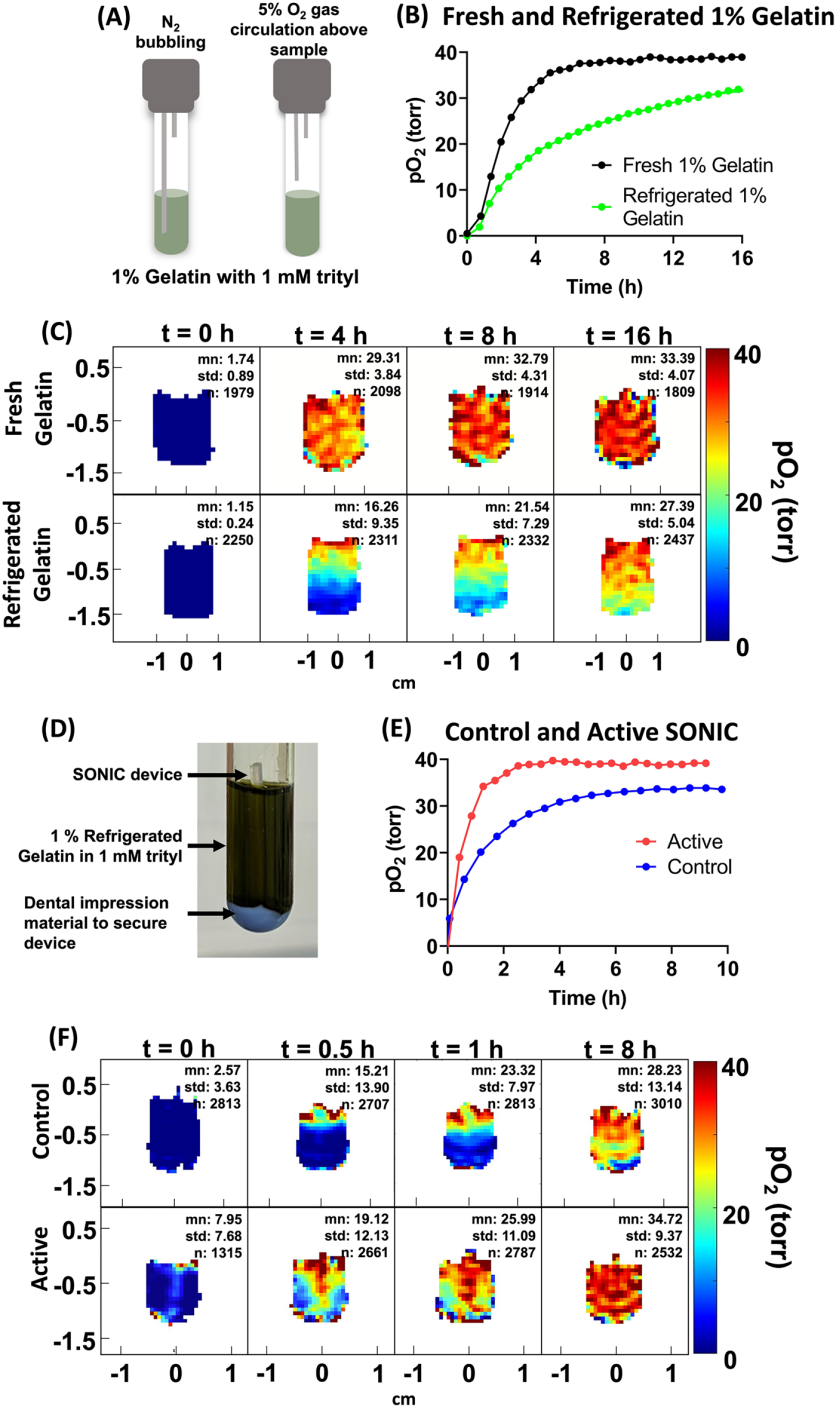
(**A**) The schematic of the experiment with fresh and refrigerated gelatin with 1 mM trityl OX071, the samples were deoxygenated by N_2_ bubbling and when completely deoxygenated in about an hour or so, the bubbling tube was pulled back, and 5% O_2_ was allowed to circulate on top of the sample, (**B**) the change in average pO_2_ for freshly prepared and refrigerated gelatin as a function of time, only reoxygenated part of the curve and images are shown here, (**C**) pO_2_ maps of fresh and refrigerated gelatin at t = 0, 4 h, 8 h, and 16 h, (**D**) A SONIC device embedded in 1% gelatin with 1 mM trityl OX071. The green color is because of the trityl OX071, (**E**) the change in average pO_2_ as a function of time for the control and the active device (n = 1), (**F**) pO_2_ maps at t = 0, 0.5 h, 1 h, and 8 h for control and active SONIC devices.

Figure 2 shows the data from trityl toxicity studies using four different cell types: fibroblast and insulin producing cells (beta-TC6, MIN6, and human islets) for 1 h, 24 h, and 48 h periods. The typical concentration of trityl used for in vitro pO_2_ imaging is 1 mM or less throughout this and other studies in the field^34,38,70,71^. We performed the toxicity study using eight concentrations from 0 (control) to 8 mM. The luminescence intensities were insignificant in the presence and absence of trityl, confirming that trityl is nontoxic for beta-TC6 (Fig. 3A), MIN6 (Fig. 3B), and human islets (Fig. 3C) up to 48 h treatment. These data suggest that trityl is safe for performing pO_2_ measurement in vitro for cell-loaded BAP devices.

The rest of the article is devoted to the pO_2_ measurements of five different BAP devices. These data are presented as proof-of-concept data and serve as proof of the capabilities of EPROI and JIVA-25™ to assist with the oxygen imaging assessment of BAP devices. Note that the pO_2_ maps are snapshots in time and represent the difference between the demand and supply. High pO_2_ in cellular devices could mean that the cells are not active or alive, while the lower pO_2_ could indicate active oxygen-consuming cells.

### Oxygen imaging of acellular SONIC (speedy oxygenation network for islet constructs) device

The SONIC devices were reported to have higher oxygen diffusion capacity because of their channel-embedding structure inspired by the tracheal anatomy of a larva of the mealworm beetle^38^. These devices were successfully employed in preclinical studies of T1D and shown to reverse hyperglycemia in mice^38^. We performed pO_2_ imaging of acellular devices to test the compatibility of EPROI to obtain the oxygen diffusion dynamics of SONIC devices. To mimic tissue conditions, the experiments were performed using 1% gelatin as the medium. To test the gelatin oxygen diffusion characteristics, as shown in the schematic of Fig. 3A, either fresh or refrigerated 1% gelatin in 1 mL volume containing 1 mM trityl in a 10 mm tube was completely deoxygenated by bubbling with 100% N_2_. Once deoxygenation was confirmed, the bubbling line was pulled to the top of the sample, and the gas was switched to 5% O_2_. The average pO_2_ values using the spectroscopic T_1_ sequence (Fig. 3B) and pO_2_ maps (Fig. 3C) using the IRESE sequence were obtained as a function of time with the overhead flow of a 5% O_2_ gas mixture throughout the experimental period. It is clear that refrigerated gelatin has a slower oxygen diffusion rate compared to freshly prepared gelatin. Our next goal was to obtain the oxygen dynamics of SONIC devices, and we used overnight refrigerated gelatin as a medium of choice because of its oxygen diffusion properties. Figure 3D shows an image of a SONIC device in a glass tube. The top of the device was exposed to air, thus allowing oxygen diffusion from the channel. Figure 3E shows the change in average oxygenation as a function of time when the control (without the channels) or active devices were placed in 1% gelatin (overnight refrigerated) and Fig. 3F shows the representative pO_2_ maps. It is clear that the active devices support rapid oxygenation compared to the control device. The gel with the active device was fully oxygenated in approx. 2 h compared to the gel with the control device, which took many hours to oxygenate.

### Oxygen imaging of acellular iBED (inverse breathing encapsulation device)

**Figure 4:**
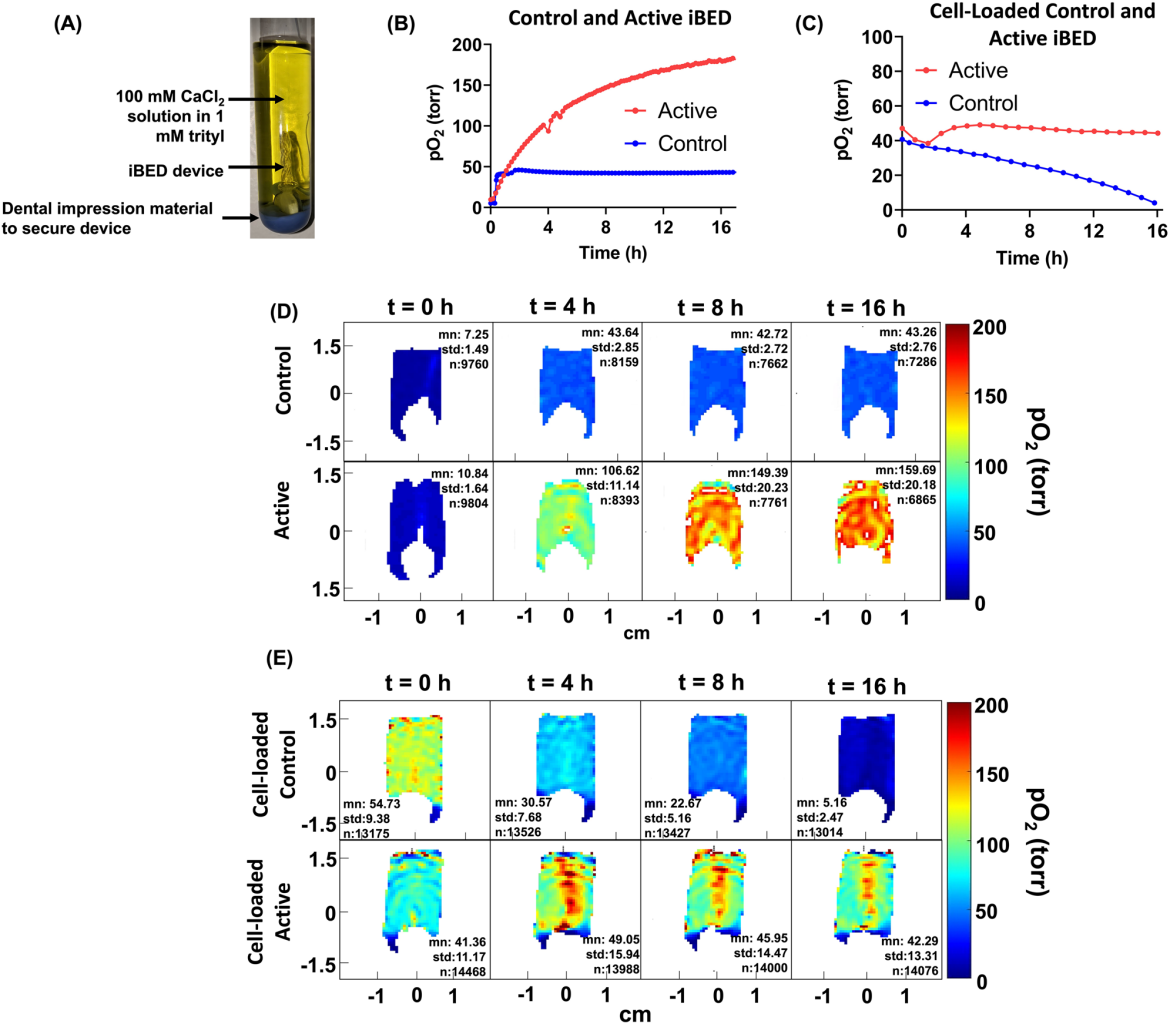
(**A**) An iBED device submerged in 100 mM CaCl_2_ solution mixed with 1 mM trityl in a 16 mm sample tube, (**B**) the change in average pO_2_ as a function of time for control and active iBED (n = 1) and (**C**) the change in average pO_2_ as a function of time (n = 1) of cell-loaded control and active devices in 1% gelatin, (**D**) selected pO_2_ maps for the active and the control devices at t = 0, 4 h, 8 h, and 16 h, (**E**) pO_2_ maps for cell-loaded samples along with iBED control and active devices in 1% gelatin. The active device could maintain higher oxygen for up to 16 h while generating oxygen that is beautifully visualized in pO_2_ maps in (**E**) (n = 1). Experimental conditions for (**B**) and (**D**): The sample tube was sealed with a septum, and N_2_ gas was bubbled at a flow rate of 10 sccm for approximately 1 h. After reaching N_2_ equilibrium, a gas mixture of 5% O_2,_ 5% CO_2_, and 90% N_2_ was bubbled at a flow rate of 6 sccm for about 1 h. Then, the gas line was raised for overhead circulation of the gas mixture for the remaining experiment at 6 sccm. These experiments were performed at room temperature. Conditions for (**C**) and (**E**): the experiment began with the bubbling of gas mixture (5% O_2_, 5% CO_2_, and 90% N_2_) at 6 sccm for a short time. The gas line was then raised to allow overhead circulation of gas at the same rate. The temperature was kept at 37 °C.

The iBED devices belong to the class of oxygen-generating devices and were reported to show their oxygen-generating capabilities and long-term diabetes reversal in immunocompetent mice implanted with islet cells^34^. The alginate-based device uses Li_2_O_2_ particles that react to the cells’ waste product CO_2_ in a self-regulated way. We validated the CO_2_-responsive oxygen-generating pO_2_ imaging of control and active devices in the presence of pO_2_ in water and gelatin. Figure 4B and D shows the change in pO_2_ as a function of time for the control and active acellular devices after deoxygenation. The gas mixture during the experiments was 5% CO_2_, 5% O_2_, and 90% N_2_ to mimic in vivo conditions. As seen from the average pO_2_ values as well as pO_2_ maps in Fig. 4B and D, the active device continues to generate oxygen for up to 16 hours.

We also performed pO_2_ measurements of the control and iBED devices seeded with 10 million fibroblast cells in 1 mL of 1% gelatin. Fig. 4C and E show the average pO_2_ for control and active devices along with pO_2_ maps at four different times during the 16-h measurement time. The active device in this experiment maintained pO_2_ at approximately 40 torr throughout the measurement, while the control device pO_2_ continued to decrease to zero during this time. The pO_2_ maps visualize the oxygen generation in the active device, as shown by the red spots in the middle.

### Oxygen Imaging of oxygen-generating agarose-based OxySite device

**Figure 5:**
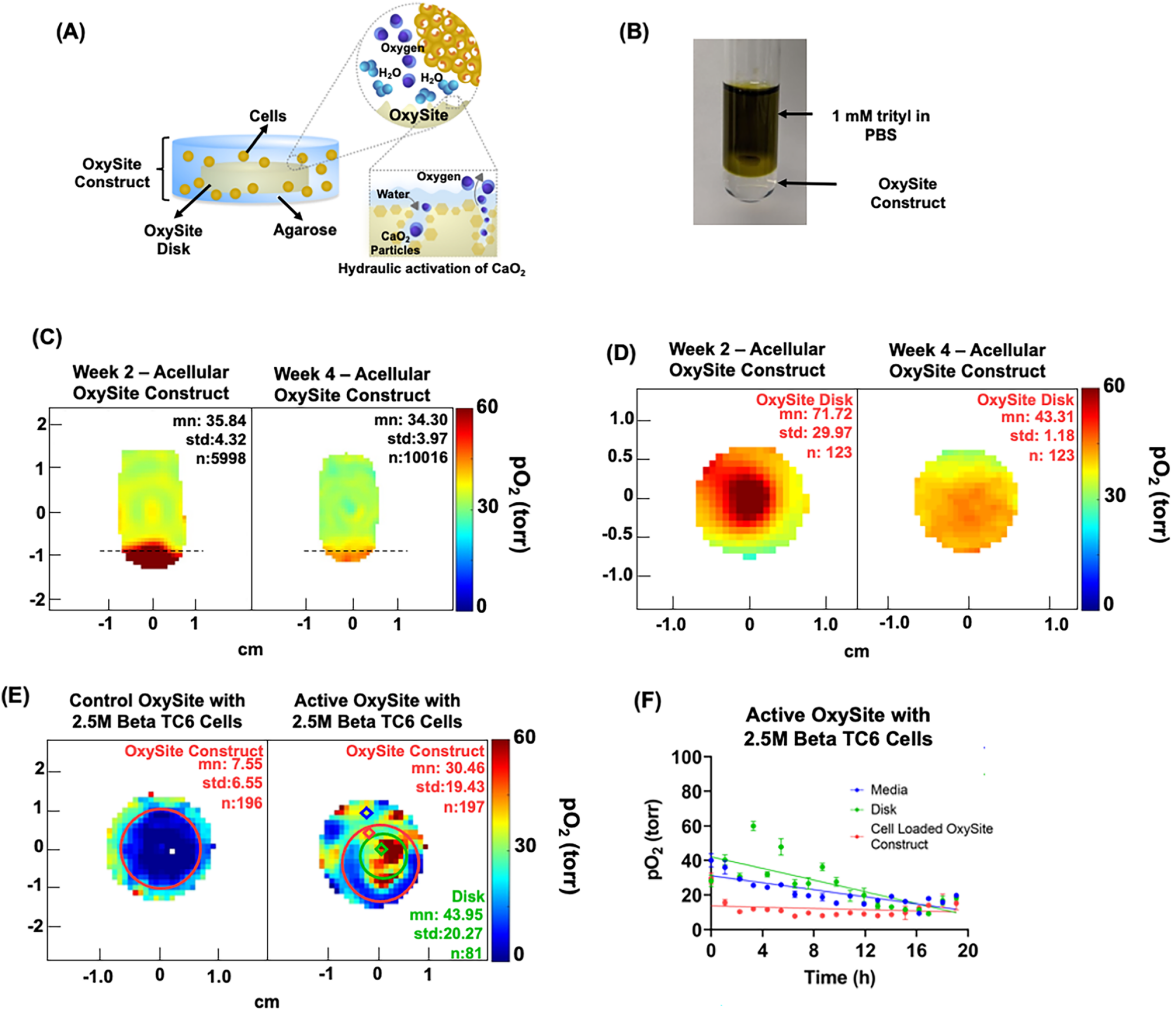
(**A**) Schematic of an OxySite construct with the oxygen-generating or control disk in the middle embedded in 2% agarose, (**B**) an active OxySite construct (in the bottom) immersed in 3 mL PBS containing 1 mM trityl in a 16 mm bottle, (**C**,**D**) The in vitro pO_2_ maps (sagittal and axial slices) of an active device submerged in PBS at week 2 and week 4. The approximate location of the axial slices is shown by dashed lines in (**C**). The system was kept closed throughout the experiment. (**D**) The pO_2_ maps of a control and an active OxySite device seeded with 2.5 million beta-TC6 cells ~ 2 h after cell seeding at 37 °C and with the circulation of 95% air and 5% CO_2_ over the media. The control device is hypoxic due to oxygen consumption of cells, while the active device had much higher oxygenation in the oxygen-generating disk and lower oxygen in the periphery, (**E**) the pO_2_ statistics as a function of time of three selected voxels from the media (blue), the disk that is generating oxygen (green), and the periphery of the device (red) as denoted by colored diamonds in pO_2_ map of the cell-seeded active construct in (**D**) (n = 1).

The agarose-based oxygen-generating OxySite devices have been shown to support islet functionality^25,32^. These agarose-based devices have an oxygen-generating disk in the middle, as shown in the schematic in Fig. 5A. The control (empty PDMS) or active (CaO_2_ particles are embedded in a PDMS) disks are placed in 2% agarose gel to create control or active OxySite constructs. We performed pO_2_ measurement of acellular constructs by placing the devices in a 16 mm sample tube filled with PBS and 1 mM trityl (Fig. 5B) to confirm the oxygen-generating capabilities of the constructs for up to four weeks, as shown in Fig. 5C (a sagittal slice in the middle of the tube) and 5D (an axial slice each for week 2 and week 4, the dashed line in Fig. 5C shows the approximate location of the axial slices). We also performed pO_2_ imaging of OxySite constructs seeded with 2.5 million beta-TC6 cells at 37 °C with 95% air and 5% CO_2_ overhead flow during the measurement. Here, we show representative pO_2_ maps (Fig. 5E) from a cell-loaded control (without an oxygen-generating disk) and a cell-loaded active device (with an oxygen-generating disk) to demonstrate the ability of EPROI to visualize the oxygen heterogeneity of such constructs. Figure 5D shows the axial pO_2_ maps of the cell-loaded control construct and the cell-loaded active construct. As we can see, the control construct is completely hypoxic, while the active construct had higher oxygen. We can see the oxygen-generating disk in the active construct pO_2_ map. Figure 5E shows the voxel kinetics from the active device pO_2_ map, in the agarose construct (red), in the oxygen-generating disk (green), and in the surrounding medium (blue) in the active device construct. The takeaway message from the images and statistics is that EPROI could easily distinguish the different areas based on oxygen, and the voxels in the constructs that were far away from the oxygen-generating disk were hypoxic.

### Oxygen imaging of TheraCyte, cell encapsulation device

**Figure 6:**
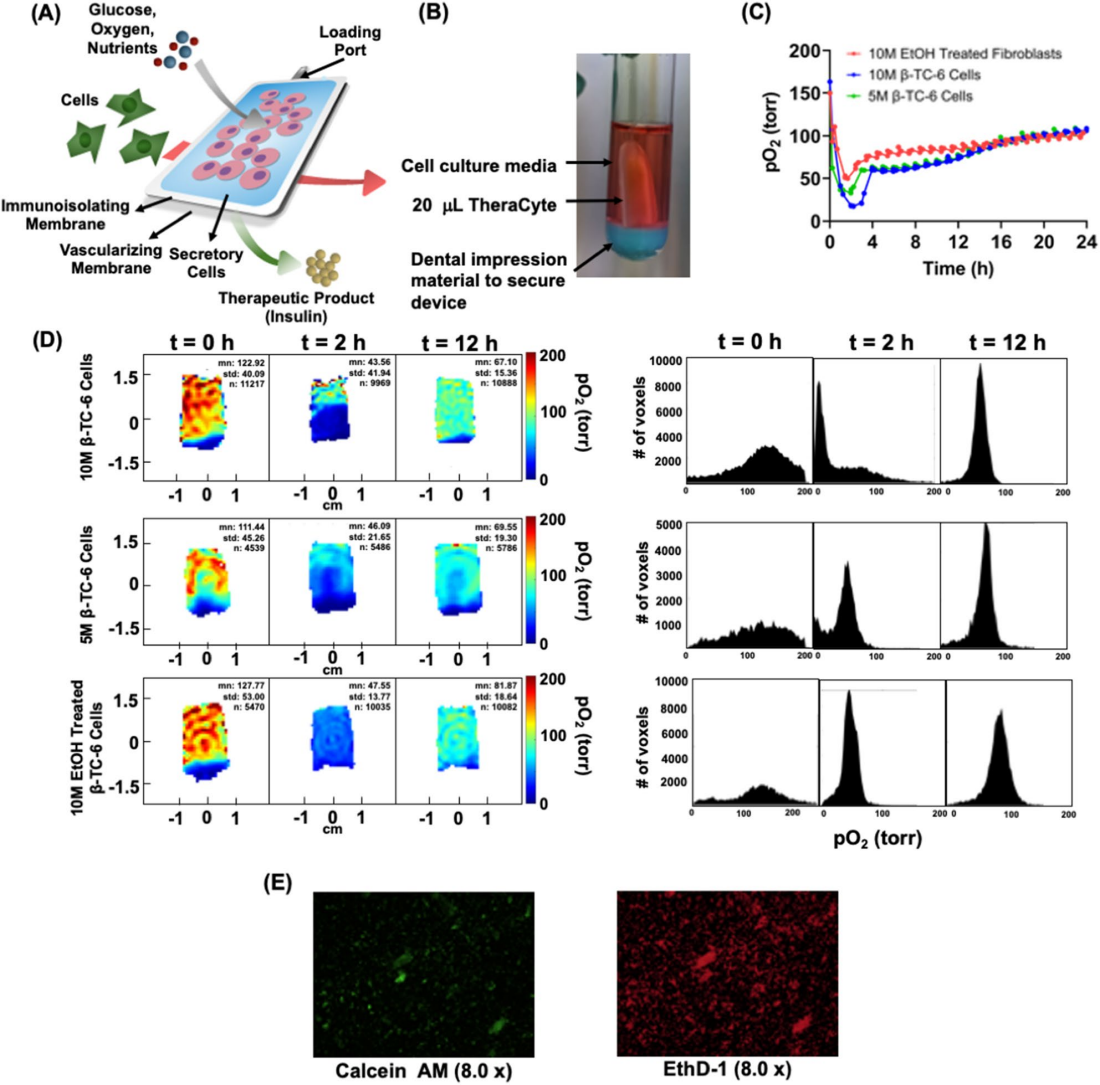
(**A**) Schematic of a TheraCyte device, (**B**) a 20 µL TheraCyte immersed in cell culture medium, (**C**) average pO_2_ as a function of time of 20 µL TheraCyte device loaded with 10 million beta-TC6 cells, 5 million beta-TC6 cells, and 10 million EtOH-treated fibroblasts (n = 1), (**D**) representative pO_2_ maps and pO_2_ histograms at t = 0, 2 h, and 12 h for the device loaded with cells and overhead flow of 95% air and 5% CO_2_ at 37 °C, (**E**) the live-dead cell assay at 24 h for the 10 million beta-TC6 cells in the device.

To assess the compatibility of EPROI with cell encapsulation devices, we performed pO_2_ imaging of a commercially available TheraCyte™ device (Fig. 6A) that has been utilized in many studies^3,29,72–76^. The device has a membrane with small enough pores to block immune cells from entering the device, thereby protecting the transplanted cells from allogeneic rejection. The device allows small molecules like glucose and oxygen to enter and be utilized by the encapsulated cells, while insulin is released by the encapsulated cells in response to glucose escapes from the device. Insulin from the device is taken up by small blood vessels surrounding it, which are formed with the help of a specially designed outer vascularizing membrane. The TheraCyte device comes in three sizes, 10 µL, 20 µL, and 40 µL. For the current experiment, 20 µL devices were chosen.

We performed pO_2_ imaging in vitro in 20 µL TheraCyte devices loaded with 5 million and 10 million beta-TC6 cells and 10 million EtOH-treated fibroblast cells. The cell-loaded 20 µL device was placed in a 16 mm tube with 3 mL of media, as shown in Fig. 6B. Throughout the experiments, a gas mixture of 95% air and 5% CO_2_ was bubbled in the media, and 37 °C temperature was maintained, and spectroscopic (average over the entire sample) pO_2_ measurements and pO_2_ images were taken every 30 min for 24 h. Figure 6C shows the average pO_2_ for all the samples, and Fig. 6D shows the representative pO_2_ maps along with the histograms at t = 0 h, 2 h, and 12 h for the device with cells. A drop in average pO_2_ values was observed for all cases. The 70% alcohol-treated fibroblast cells also show a drop in pO_2_. Some published reports suggest the preservation effect of alcohol in cells, and it is possible that these cells became metabolically active when placed in media in incubator-like conditions that could have been attributed to the initial drop in pO_2_^77–79^. The pO_2_ recovered over the course of 24 h with cell viability assay on 10 million beta-TC6 cells loaded device (Fig. 6E) showed that most of the cells were not alive at 24 h.

### Oxygen imaging of a perfused macro-encapsulation device

**Figure 7:**
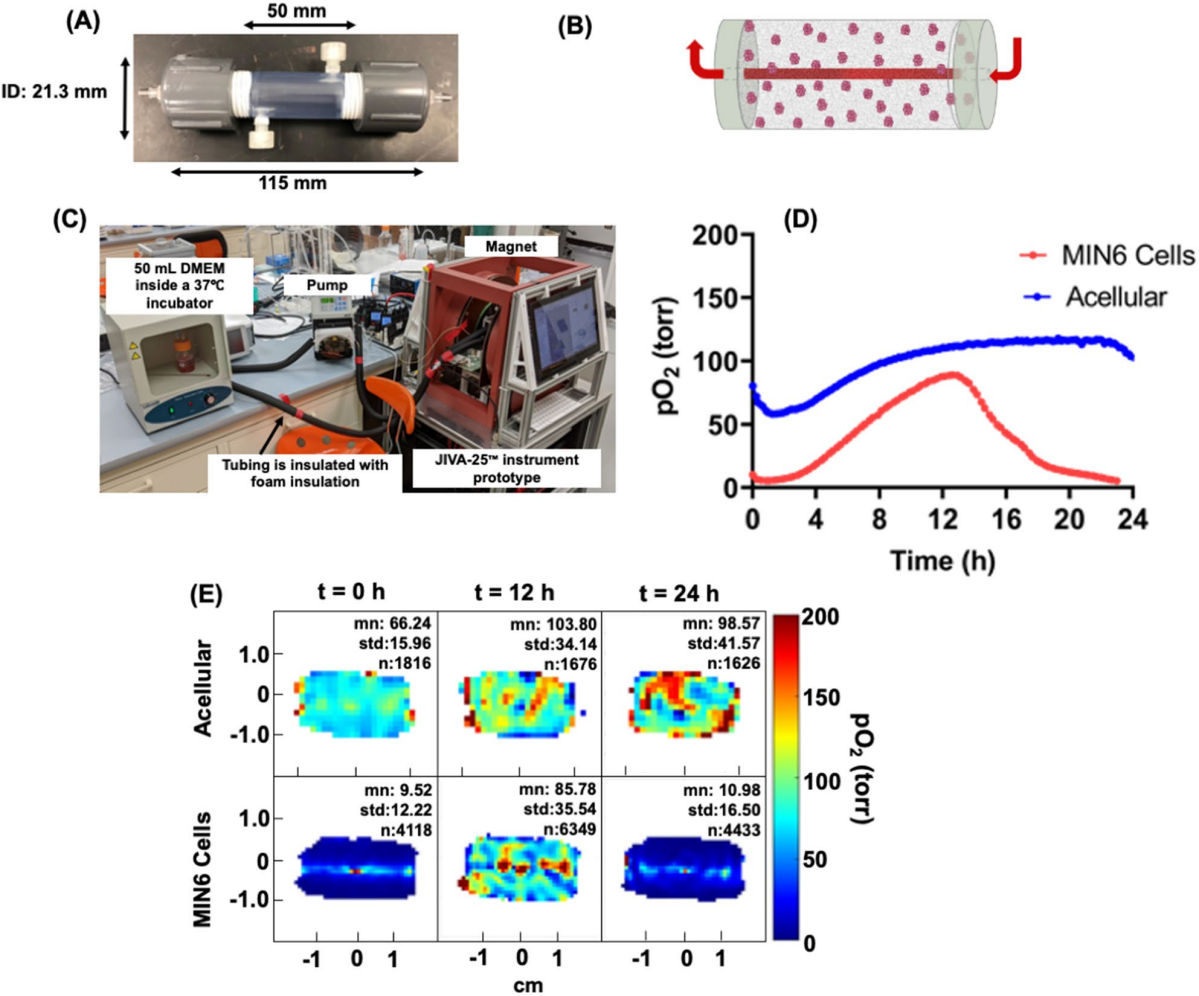
(**A**) An image of the 50 mm long macro-encapsulation device, (**B**) schematic representation of the device showing a channel in the middle, (**C**) the set-up for pO_2_ imaging, the device was set up inside the 20 mm × 25 mm resonator, and the bottle containing media was kept in a small incubator at 37 °C in a small incubator. The media was circulated through the insulated tubing that passed through the device and back with the support of a peristaltic pump, (**D**) the average pO_2_ as a function of time in an acellular and 24 million MIN6 cell loaded device (n = 1) and (**E**) representative pO_2_ maps of acellular (top panel) and cell loaded (bottom panel) devices at t = 0, 12 h, and 24 h.

As a final example of pO_2_ imaging of BAP, we performed pO_2_ measurement in a large-size macro-encapsulation device (Fig. 7A). The device was designed to accommodate therapeutic islet doses (600,000) in multi-channel devices^65,66^, but a single-channel system was applied here to illustrate the oxygen gradients established in this simplified case. This alginate-based device was developed using internally gelled alginate, and a channel was created in the middle, as shown in the device schematic in Fig. 7B^65^. Because of the large size of the device, a horizontal resonator with a 20 mm diameter and 25 mm length that could accommodate the middle section of the device for the measurements was developed. To keep the channel flowing with the warm media, a beaker filled with media was kept in a small incubator at  37 °C which was placed it near the instrument, as shown in Fig. 7C. This beaker was connected with insulated tubing to the device. We performed pO_2_ imaging of acellular and 24 million MIN6 cells- (1.2 million per mL) loaded devices for 24 h. Figure 7D provides the change in average pO_2_ across the device in both cases, and Fig. 7E provides pO_2_ maps for the selected time points (t = 0, 12 h, and 24 h). Interestingly, initial pO_2_ was lower than expected in both acellular (~ 90 torr), and the cell-loaded (~ 10 torr) device became quickly hypoxic, potentially due to CO_2_ generation during CaCO_3_ conversion to Ca^2+^ and H_2_CO_3_ (equilibrium with HCO_2_^-^ and CO_2_ forms). As media continues to flow, the pO_2_ slowly increased in both devices. In the case of the acellular device, it plateaued at around 120 torr, while in the cell-loaded device, it increased up to about 80 torr before decreasing again to the hypoxic level at 24 h. The increase in average pO_2_ followed by a subsequent decrease could be due to the CO_2_ partial pressure effect dissipating during medium perfusion, followed by gradual consumption of the oxygen “reservoir” in the initial bulk gel by the MIN6 cells thereafter. After 24 h, the pO_2_ profiles and values as a function of distance from the channel were close to expected values from numerical models at this MIN6 seeding density^66^.

## Conclusion and discussion

The current feasibility studies of in vitro oxygen imaging with commonly used biomaterials, such as alginate, gelatin, agarose, etc., and five BAP devices show that the EPROI provides average pO_2_ in samples as well as pO_2_ maps with high spatial and pO_2_ resolution in these devices. The experiments with fresh and refrigerated gelatin provide three-dimensional oxygen diffusion characterization of these biomaterials. We hope that this data will prompt scientists to characterize their biomaterials for oxygen diffusion characteristics. Trityl OX071 toxicity study shows that trityl is nontoxic to cells. The comparison of EPROI with OxyLite Pro demonstrate that EPROI is highly precise in providing pO_2_ measurements. The pO_2_ imaging of five BAP devices as a function of time, SONIC, iBED, OxySite, TheraCyte, and perfused single channel macro-encapsulation device, show that the EPROI technique is robust and that the devices and cells can be observed longitudinally. We present EPROI as a quality control tool for developing efficient BAP devices. We expect that longitudinal pO_2_ mapping of BAP devices will have many advantages: (a) scientists can choose to intervene if hypoxia is visible in pO_2_ maps, (b) scientists can perform interventions such as additional oxygen supplies and additional islet transplantation to obtain optimized device performance, (c) pO_2_ mapping can guide device geometry optimization efforts, and (d) pO_2_ maps can guide how to optimize the device performance based on the oxygen distribution in the device, and (e) pO_2_ maps can be utilized to optimize the cell seeding density in BAP devices. These benefits make pO_2_ imaging an essential tool for islet transplantation therapy. In addition to T1D, in  vitro pO_2_ mapping technology could also be useful for the quality control of artificial tissue grafts and organs. We hope that oxygen imaging will be utilized widely by the tissue engineering regenerative medicine community in the coming years and will become a standard quality control tool in the field that will eventually provide an efficient cure for T1D and many other medical conditions.

## Data Availability

The datasets used and analyzed during the current study are available from the corresponding author upon reasonable request.
